# Predictors of Internet Use Among Older Adults With Diabetes in South Korea: Survey Study

**DOI:** 10.2196/19061

**Published:** 2020-12-23

**Authors:** Sunhee Park, Beomsoo Kim

**Affiliations:** 1 Barun ICT Research Center Yonsei University Seoul Republic of Korea; 2 Graduate School of Information Yonsei University Seoul Republic of Korea

**Keywords:** digital divide, internet use, older adults, diabetes, health, internet, Korea

## Abstract

**Background:**

Internet access in Korea has grown dramatically over the past two decades. However, disparities in internet use, referred to as the second level of the digital divide, persist.

**Objective:**

This study aims to examine opportunity, motivation, and health variables that indicate internet use among older adults with diabetes.

**Methods:**

Data were sourced from a nationally representative sample of people 65 years and older with diabetes (N=1919). Logistic regression was used to explore potential differences in predictor variables between internet users and nonusers.

**Results:**

Only 306 of the 1919 (15.95%) participants in the sample used the internet. They were more likely to be younger (odds ratio [OR] 0.89, 95% CI 0.87-0.92), well-educated (OR 1.20, 95% CI 1.16-1.26), and able to afford leisure expenditures (OR 1.02, 95% CI 1.01-1.04). Additionally, they had more information and communications technology (ICT) training experience, were motivated to learn, volunteered, and reported good physical and cognitive function. Participation in ICT education and better health more positively correlated with a higher rate of internet use than did years of education or economic standing in older adults with diabetes.

**Conclusions:**

To support older adults with diabetes in the internet age, policies and health care providers should focus on digital competency training as well as physical and cognitive function.

## Introduction

Internet access has grown dramatically over the past two decades in Korea. However, disparities in internet use still persist [1,2]. This disparity is known as the second level of the digital divide, which refers to a gap in access (the first level), use (the second level), and outcomes (the third level) of information and communications technology (ICT). Digital competency enables older adults to live more convenient lives and plays an important role in maintaining quality of life, health care, independent living, and relationships and in reducing isolation [3,4].

With a rapid increase in Korea’s older adult population, in which chronic diseases are prevalent, addressing aging-related problems is important [5]. Diabetes mellitus is one of the most common chronic diseases affecting lifestyle, and its prevalence is increasing worldwide. In Korea, 25.1% of older adults 65 year and older have diabetes, and their mortality rate due to diabetes or cerebrovascular disease is higher than the Organisation for Economic Co-operation and Development average, partly because of the vulnerability related to preventing deaths from treatable conditions [2]. An unhealthy lifestyle contributes to diabetes to a great extent, and one of the mainstays of diabetes treatment and prevention is adopting a healthy lifestyle. As there is no cure for diabetes, recently, self-management by mobile health or eHealth has begun to play a vital role in the digital era.

Many systematic reviews and meta-analyses have indicated that eHealth tools are effective in self-management both for disease management and lifestyle changes in daily life [5-7], and limited internet use and low eHealth literacy can indirectly cause health problems [8]. Problems with eHealth literacy due to low cognitive function make it difficult for older adults to manage, prevent, and treat diseases. This in turn leads to health problems [9], poor management of chronic diseases [10], and lower participation in treatment interventions. Low eHealth literacy is also associated with medical service misuse, which can be fatal [11]. Furthermore, the second digital divide, the gap in internet use, alienates older adults, leading to losses in self-employment opportunities, social exchanges, advantageous purchases, and investments. It also contributes to health problems caused by social network loss [12].

Internet underutilization by older adults is due primarily to limited opportunity and motivation [13]. Limited opportunity affects individuals who do not access the internet due to socioeconomic problems or lack of information. In a study of urban dwellers, only 27% of older adults were found to use computers, and age, years of education, occupation, income level, self-rated health, and volunteer work were the affecting factors [14]. Limited motivation indicates individuals who have not voluntarily chosen internet use and do not accept new technologies because they have no incentive or interest in them. In general, older adults lack ICT knowledge and skills and are often unaware of the need for it [15]. Moreover, older adults lack the confidence or support needed to learn how to use new equipment or acquire new knowledge. This low intention to acquire new knowledge results in a low level of internet use [16]. In addition to opportunities and motivation, aging and health problems involving physiological and cognitive functions also determine internet use, as do daily activities and chronic diseases [17,18]. Internet use has increased in Medicare-eligible patients but remains very low among the frailest older adults. Therefore, functional ability is more indicative of internet avoidance than chronic illness, self-rated health, or age [19].

Barriers to access and use include financial restrictions (ie, equipment and subscription costs are too high), medical and disability-related constraints (ie, the technology is not accessible or intuitive), and digital complexity (ie, accessing and navigating the internet is too complex) [20]. Scheerder et al [21] systematically reviewed 126 papers and distinguished 7 factors contributing to the digital divide: demographics, economics, social networks, cultural context, physical activity, home access and device availability, and attitudes toward online technology. Leisure activity and voluntary work were the affecting factors of internet use, and low levels of internet use affected social networking [12,21].

Although internet use among older adults is less prevalent than in the general public and is associated with aging or health problems [22], some older adults, such as those in the baby boomer generation, use the internet effectively because they are highly educated and were gradually exposed to smartphones and digital devices [20]. They use the internet to search for health-related information and exhibit confidence and satisfaction regarding eHealth [23].

**Figure 1:**
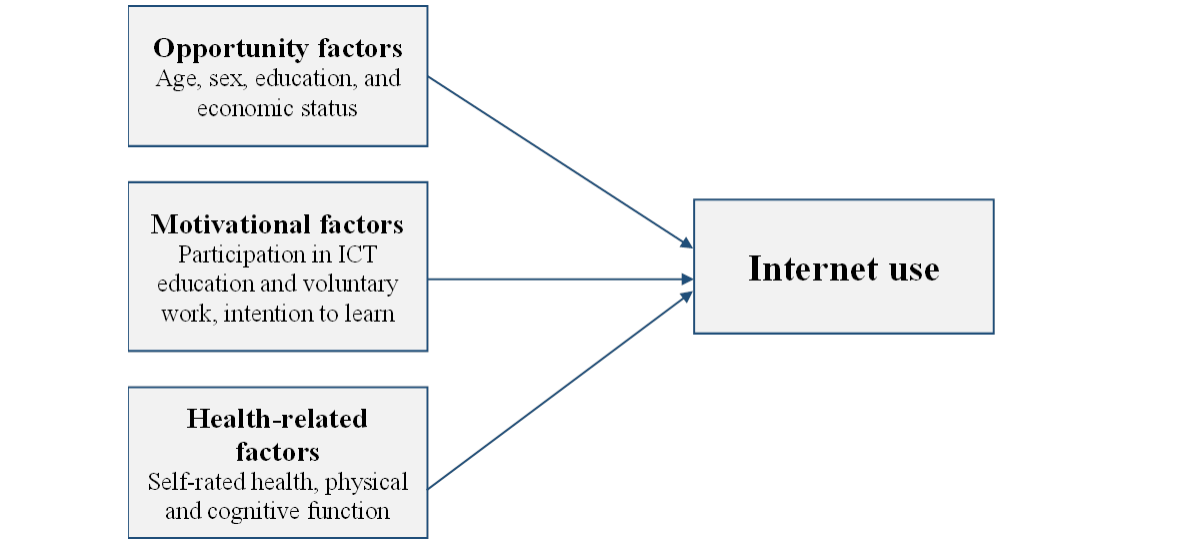
Factors related to internet use. ICT: information and communications technology.

As older adults are vulnerable to aging-related issues and chronic diseases, studies of internet usage among older adults with health problems or chronic diseases are needed. Furthermore, there is limited information on the predictors of internet use among older adults with diabetes, a chronic disease that demands continuous lifestyle modification and self-care. The aim of this study was to examine opportunity, motivation, and health-related factors that determine internet use among older adults with diabetes in South Korea (Figure 1).

## Methods

### Design and Sample

The data for this study came from the 2017 Survey of Living Conditions and Welfare Needs of Korean Older Persons from the Korea Institute for Health and Social Affairs, which was based on a nationally representative sample of participants 65 years and older who were recruited using a stratified 2-stage cluster sampling design. The survey collected information through face-to-face interviews, and all participants provided written informed consent [2]. The sample for this study included 1919 (of 10,299) respondents with diabetes who reside in the community. The inclusion criteria were age of 65 years or older, official diagnosis of diabetes for more than 3 months with treatment, and response to a survey on internet use. We excluded individuals who did not respond to the survey on internet use and those younger than 65 years. The design was considered exempt from ethical review by the institutional review board of Yonsei University (approval no. 7001988-202001- HR-777-01E), as the data were anonymized.

### Measurements

Internet use was assessed with 1 item: the use or nonuse of the internet or mobile phones to browse for information. Participants were asked, “Is it possible for you to use smart phones, computers, tablet PCs, and internet television to search for information?” to which they answered with either “yes” or “no.” Participants provided demographic and socioeconomic information such as age, sex, and years of education. Leisure activity expenditure was assessed according to monthly average expenditure on leisure activities in Korean won to determine participants’ economic standing [24]. Having previous or current volunteer experience was classified as either “yes” or “no.” Participation in ICT education was assessed by the question “Have you participated in ICT education during the last five years?” Participants responded with either “yes” or “no.” Intention to learn was measured on a 5-point Likert scale (1=no intention; 5=very eager to learn). Health-related factors included self-rated health, physical function, and cognitive function. Self-rated health was assessed by one question: “How do you feel about your health?” It was scored from 1 to 5 (1=not good at all; 5=very good). The higher the number, the higher the self-rated health score. Physical function was assessed using the 11-item Korean Instrumental Activities of Daily Living (K-IADL) questionnaire (ability to use a telephone, go shopping, prepare food, perform housekeeping and laundry, handle medication and finances, use transportation, and drive); total scores range from 11 and 33. The higher the score on the K-IADL, the lower the physical function [25]. A score of 33 on the K-IADL represents physical dependency. Cognitive function was assessed using the Korean version of the Mini-Mental State Examination for Dementia Screening (MMSE-DS); total scores range from 0 to 30. The higher the score, the better the cognitive function [26].

### Data Analysis

Stata 15.1 (StataCorp) was used to conduct data analyses. Univariate analyses were performed to identify associations between internet use and factors related to opportunity, motivation, and health. Independent variables with significant group differences in the univariate analyses were included in a multivariate logistic regression analysis, which was performed to calculate the adjusted odds ratios (ORs) for internet users and nonusers.

## Results

Of the 1919 respondents, only 306 (15.95%) used the internet to search for information (Table 1). Internet users were more likely to be male, younger, and more educated; have a higher leisure activity expenditure; volunteer more; have ICT education experience; have a lower intention to learn; and have better self-rated health, physical function, and cognitive function than internet nonusers.

**Table 1:** Participant characteristics (N=1919).

Characteristics and variables			Total	Internet users	Internet nonusers	*t* test^a^ (*df*)	*F* test (*df*)	*P* value
**Dependent variable**								
	Internet use, n (%)		1919 (100.0)	306 (16.0)	1613 (84.0)	N/A^b^	N/A	N/A
**Opportunity factors**								
	Age (years), mean (SD)^c^		75.15 (5.66)	71.96 (0.27)	75.75 (0.14)	11.06 (1)	N/A	<.001
	**Gender, n (%)**					N/A	64.27 (1)	<.001
		Male	1312 (68.4)	269 (87.9)	1043 (64.7)			
		Female	307 (31.6)	37 (12.1)	570 (35.3)			
	Education (years), mean (SD)^d^		7.40 (4.64)	11.06 (0.21)	6.71 (0.11)	–16.03 (1)	N/A	<.001
	Leisure expenditure (in ₩10,000)^e^, mean (SD)		6.08 (11.00)	14.18 (1.06)	4.55 (0.20)	–14.83 (1)	N/A	<.001
**Motivational factors**								
	**Participation in ICT^f^ education, n (%)**					N/A	41.98 (1)	<.001
		Yes	16 (0.8)	12 (3.9)	4 (0.3)			
		No	1903 (99.2)	294 (96.1)	1609 (99.7)			
	Intention to learn, mean (SD)		2.01 (0.98)	3.49 (0.06)	4.09 (0.02)	9.96 (1)	N/A	<.001
	**Voluntary work, n (%)**					N/A	84.36 (1)	<.001
		Yes	290 (15.1)	99 (32.4)	191 (11.8)			
		No	1629 (84.9)	207 (67.6)	1422 (88.2)			
**Health-related factors**								
	Self-rated health, mean (SD)^g^		2.57 (0.92)	2.97 (0.05)	2.49 (0.02)	–8.50 (1)	N/A	<.001
	K-IADL^h^ dependency, mean (SD)		11.12 (2.42)	10.21 (0.06)	11.29 (0.06)	7.30 (1)	N/A	<.001
	MMSE-DS^i^, mean (SD)		24.94 (3.78)	27.47 (0.13)	24.46 (0.10)	–13.32 (1)	N/A	<.001

^a^2-tailed *t* tests.

^b^N/A: not applicable.

^c^Age range was 69 to 95 years.

^d^Education range was 0 to 20 years.

^e^A currency exchange rate of ₩1084.74=US $1 is applicable.

^f^ICT: information and communications technology.

^g^Self-rated health range was 1 to 5.

^h^K-IADL: Korean Instrumental Activities of Daily Living (range of 11-33**).**

^i^MMS-DS: Mini-Mental State Examination for Dementia Screening (range of 0-30).

Prior to multivariate logistic regression, multicollinearity was assessed and the variance inflation factor of all the individual variables did not exceed 10.0 (1.06-2.10). The logistic regression analysis (Table 2) revealed that internet use was independently associated with younger age (OR 0.89, 95% CI 0.87-0.92), higher educational level (OR 1.20, 95% CI 1.16-1.26), and higher leisure activity expenditure (OR 1.02, 95% CI 1.01-1.04). Internet users had more experience with ICT education and were more motivated to learn than nonusers. The ORs showed that the odds of participation in ICT education were about 10 times higher (OR 9.75, 95% CI 2.39-39.84) and the odds of voluntary work were over 2 times higher (OR 2.09, 95% CI 1.48-2.94) for internet users compared with nonusers. Users were also more likely to have better K-IADL scores (OR 0.78, 95% CI 0.66-0.92), higher MMSE-DS scores (OR 1.19, 95% CI 1.12-1.27), and better perceived health status (OR 1.27, 95% CI 1.08-1.50).

**Table 2:** Logistic regression model predicting internet use among older adults with diabetes mellitus (N=1919).

Characteristics and variables		OR^a^ (95% CI)	*P* value
**Opportunity factors**			
	Age (years)	0.89 (0.87-0.92)	<.001
	Education (years)	1.20 (1.16-1.26)	<.001
	Leisure expenditure (₩)	1.02 (1.01-1.04)	<.001
**Motivational factors**			
	Participation in ICT^b^ education (reference: none)	9.75 (2.39-39.84)	.002
	Intention to learn	1.39 (1.20-1.60)	<.001
	Voluntary work (reference: no)	2.09 (1.48-2.94)	<.001
**Health-related factors**			
	Self-rated health	1.27 (1.08-1.50)	.004
	K-IADL^c^ dependency	0.78 (0.66-0.92)	.003
	MMSE-DS^d^ (score)	1.19 (1.12-1.27)	<.001

^a^OR: odds ratio.

^b^ICT: information and communications technology.

^c^K-IADL: Korean Instrumental Activities of Daily Living.

^d^MMSE-DS: Mini-Mental State Examination for Dementia Screening.

## Discussion

### Principal Findings

This study attempted to provide basic data on indicators of internet use among older adults with diabetes in South Korea by identifying relevant variables related to opportunity, motivation, and health. Age, years of education, economic standing, ICT education, volunteer experience, physical function, and cognitive function were identified as major predictors of ICT use among older adults with diabetes.

Only 15.95% (306/1919) of the participants used the internet to search for information in this study. In South Korea, 38.5% of people aged 60 to 89 years use ICT [27]. A study on US residents showed that 27% of urban residents used computers and 38% of patients receiving kidney transplants used the internet [13,28]. These results are in line with studies showing that older adults with chronic diseases use the internet less than younger populations [22]. Some studies have shown that individuals frequently use the internet to search for health information, even when patients had chronic diseases [28]. It is necessary to exercise caution in interpreting whether chronic diseases predict internet use. In this study, more than 80% (1613/1919, 84.05%) of the participants did not use the internet, indicating a need for social policies to bridge the digital divide and improve internet use among older adults with diabetes.

Internet use among older adults is closely related to age, sex, and years of education [29], and the same results were demonstrated for the older adults with diabetes in this study; age was a predictor of internet use in older adults with diabetes.

In Korea, internet access has grown over the past two decades (Multimedia Appendix 1). Over 90% of the population has internet access through national support and various policies [1]. In this study, according to the leisure activity expenditure, the economic predictor of internet use signifies that a digital divide still exists among older adults with diabetes. Therefore, it is important to approach the digital divide in older adults with diabetes from the perspective of accessibility.

Participation in ICT education can be a possible predictor of internet use among older adults with diabetes. This result was in line with previous research, which found that older adults who knew how to use computers before they were 65 years old were 9 times more likely to use the internet than those who did not [30]. Therefore, the capabilities of using the internet and the ICT skills of older adults with diabetes should be assessed by health care providers prior to digital interventions or individualized education programs.

The focus of research on the digital divide has recently shifted from accessibility to utilization and outcomes. Many studies have shown that personal preferences and motivations, in addition to opportunities and structural aspects, influence active internet use [20]. This study revealed that internet nonusers were more willing to receive information on service education. It could thus be inferred that internet nonuse correlates with fewer technology training opportunities and that more training is needed for frail older adults and their caregivers to effectively use the internet to engage in care [24]. Therefore, individualized education programs for older adults with diabetes should include disease-related and ICT education.

In this study, volunteer activities as a type of social participation or activity predicted internet use. The results are consistent with studies that show that internet or mobile phone use by older adults is strongly related to social activities, social support, and self-esteem [27]. Leisure activity expenditure is a good proxy for economic status [24] and was a good predictor of internet use among older adults with diabetes in this study. Oh [31] encouraged leisure activities among older adults, such as shopping and watching entertainment shows and performances, cultural activities, videos, and movies, because these activities significantly influenced active internet use and search capabilities among older generations. It is necessary to encourage older adults with diabetes to engage in leisure and hobby activities because it may improve their digital health literacy.

In this study, physical and cognitive function were identified as predictors of internet use; internet use decreases when health and instrumental activities of daily living are degraded by physical function [19]. Instrumental activities of daily living require high levels of physical function in everyday behavior to live independently and indicate the possibility of returning to society [25]. The results showed that K-IADL score is a predictor of internet use. Having good physical functional status could encourage older adults with diabetes to participate in social activities, making them more likely to have a chance to use the internet in society [22]. Thus, functional limitations should be considered in strategies to reduce the digital divide among older adults with chronic diseases.

Cognitive function was one of the predictors of internet use among older adults with diabetes. With age, adults experience a decline in both cognitive and physical function and become restricted in activities such as delicate muscle movement, reading, and interpreting large quantities of information. Internet use requires extensive cognitive information processing and learning and can therefore burden older adults [32]. Thus, developing functions and programs that can be more easily accessed and handled by older adults with reduced cognitive function is essential in enhancing internet use and reducing the digital divide.

### Implications

Although the digital divide can be defined based on various aspects, such as access, usability, and utilization, this study focused on predictors of internet use among older adults with diabetes. We expect that improved internet use will improve self-care among this population; however, there is still a gap in internet use due to economic, social, physical, and cognitive factors [6]. In the current information age, health care systems are increasingly embracing eHealth and digital services. South Korea has created a national patient portal to provide health information through electronic devices. Meanwhile, other countries have developed digital aids using health-related applications, virtual reality, and games [33]. The weaknesses and strengths among older adults with diabetes should be properly identified to assist in the creation of individualized mediation plans. This will prevent the digital divide from separating older adults with diabetes from digital health care trends.

Due to the limitations of secondary data analysis, this study did not reflect the characteristics of the participants’ diabetes, so future research should include the relationship between diabetes characteristics and internet use. Another limitation of this study is that although it used nationally representative data, there may be errors in generalization due to the small number of participants; therefore, it is necessary to be cautious when interpreting the results.

### Conclusions

Internet use has dramatically increased in South Korea during the past two decades but remains very low among older adults with diabetes. Our results suggest that years of education, leisure activity expenditure, participation in education, intention of education, voluntary work, self-rated health, and MMSE-DS scores were positively correlated predictors of internet use, while age and K-IADL dependency were negatively correlated predictors of internet use. While prior studies of the digital divide in health care have highlighted demographics and socioeconomic status, our study demonstrates the additional impact of motivational factors and health-related factors in older adults with diabetes. Health care providers need to formulate digital health interventions to prevent the most frail and vulnerable older adults from being left out of consideration in online patient portals and eHealth. Policies and health care providers should focus on digital competency training and volunteer activities among older adults with diabetes. For functionally limited older adults, user-friendly digital aids may improve internet use. For cognitively impaired older adults, caregivers or family members should be included in the intervention. Future studies should examine more strategies to reduce the digital divide among older adults with diabetes.

## Appendix

Multimedia Appendix 1 Supplementary table.

## Abbreviations

ICT: information and communications technology
K-IADL: Korean Instrumental Activities of Daily Living
MMSE-DS: Mini-Mental State Examination for Dementia Screening
OR: odds ratio
