# Supplementary material for: Predictors of Internet Use Among Older Adults With Diabetes in South Korea: Survey Study
Source: JMIR Med Inform. 2020 Dec 23;8(12):e19061. doi: 10.2196/19061 (PMC7787888; doi:10.2196/19061)
Supplement: Multimedia Appendix 1 [file medinform_v8i12e19061_app1.docx]

Table 2 shows the univariate analyses for Internet use.

Table 2. Univariate logistic regression of internet use among older adults with diabetes mellitus

N=1919

| **Variables** | **Categories** | ^†^**OR** | **95%** ^§^**CI** | | **p** |
| --- | --- | --- | --- | --- | --- |
|  |  |  | **Lower** | **Upper** |  |
| **Opportunity factors** | Age (years) | 0.87 | 0.85 | 0.9 | <.000 |
|  | Gender (ref. male) |  |  |  |  |
|  | Female | 0.25 | 0.18 | 0.36 | <.000 |
|  | Education (years) | 1.27 | 1.23 | 1.31 | <.000 |
|  | Leisure expenditure (10,000 Korean won) | 1.07 | 1.06 | 1.08 | <.000 |
| **Motivational factors** | Participation in ICT education (ref. no) |  |  |  |  |
|  | Yes | 16.42 | 5.26 | 51.25 | <.000 |
|  | Intention to learn | 1.74 | 1.55 | 1.95 | <.000 |
|  | Voluntary work (ref. no) |  |  |  |  |
|  | Yes | 3.56 | 2.68 | 4.73 | <.000 |
| **Health-related factors** | Self-rated health | 1.74 | 1.52 | 1.99 | <.000 |
|  | ^†^IADL dependency | 0.61 | 0.53 | 0.71 | <.000 |
|  | ^§^MMSE | 1.4 | 1.33 | 1.48 | <.000 |

^†^IADL=Instrumental activities of daily living; ^§^MMSE=Mini-Mental State Exam
